# Effect of Relative Arrangement of Cationic and Lipophilic Moieties on Hemolytic and Antibacterial Activities of PEGylated Polyacrylates

**DOI:** 10.3390/ijms161023867

**Published:** 2015-10-09

**Authors:** Ashish Punia, Kevin Lee, Edward He, Sumit Mukherjee, Andrew Mancuso, Probal Banerjee, Nan-Loh Yang

**Affiliations:** 1 Department of Chemistry and Center for Engineered Polymeric Materials, College of Staten Island, Staten Island, New York, NY 10016, USA; E-Mail: apunia@gradcenter.cuny.edu; 2 Ph. D. Program in Chemistry at the Graduate Center of the City University of New York, New York, NY 10016, USA; 3 Department of Chemistry, Macaulay Honors College, New York, NY 10016, USA; E-Mails: kevin.lee@macaulay.cuny.edu (K.L.); edward.he@macaulay.cuny.edu (E.H.); 4 Ph.D. Program in Biochemistry at the Graduate Center of the City University of New York, New York, NY 10016, USA; E-Mails: smukherjee@gradcenter.cuny.edu (S.M.); amancuso@gradcenter.cuny.edu (A.M.); 5 Department of Chemistry and Center for Developmental Neuroscience, College of Staten Island, Staten Island, New York, NY 10016, USA; E-Mail: probal.banerjee@csi.cuny.edu

**Keywords:** antimicrobial polymers, amphiphilic polymers, polyacrylates, drug-resistant bacteria, PEGylated, bactericidal agents

## Abstract

Synthetic amphiphilic polymers have been established as potentially efficient agents to combat widespread deadly infections involving antibiotic resistant superbugs. Incorporation of poly(ethylene glycol) (PEG) side chains into amphiphilic copolymers can reduce their hemolytic activity while maintaining high antibacterial activity. Our study found that the incorporation of PEG has substantially different effects on the hemolytic and antibacterial activities of copolymers depending on structural variations in the positions of cationic centers relative to hydrophobic groups. The PEG side chains dramatically reduced the hemolytic activities in copolymers with hydrophobic hexyl and cationic groups on the same repeating unit. However, in case of terpolymers with cationic and lipophilic groups placed on separate repeating units, the presence of PEG has significantly lower effect on hemolytic activities of these copolymers. PEGylated terpolymers displayed substantially lower activity against *Staphylococcus aureus* (*S. aureus*) than *Escherichia coli* (*E. coli*) suggesting the deterring effect of *S. aureus*’ peptidoglycan cell wall against the penetration of PEGylated polymers. Time-kill studies confirmed the bactericidal activity of these copolymers and a 5 log reduction in *E. coli* colony forming units was observed within 2 h of polymer treatment.

## 1. Introduction

The global health threat from infections involving antibiotic resistant bacteria (superbugs) have created a pressing need to develop new antibacterial agents [1,2]. Antimicrobial resistance to antibiotics causes thousands of deaths and adds billions of dollars to health care budget in the Unites States alone [3,4,5]. It has been recently estimated that antimicrobial resistance will result in 10 million annual deaths by 2050 [6]. The problem of antibiotic resistance is further compounded by sharp decline in number of new antibiotics introduced in market in recent years due to high costs involved in their development facing the rapid development of microbial resistance against antibiotics [1]. Synthetic cationic amphiphilic polymers mimicking the principle features of natural antimicrobial peptides (AMPs) have emerged as promising candidates to fight against superbugs [7,8]. AMPs and their synthetic mimics are known to disrupt the negatively charged bacterial cell surface through electrostatic and lipophilic interactions [9,10,11]. This mechanism of bacterial cell surface rupture through non-specific interactions leads to highly hindered or improbable development of bacterial resistance, which is in contrast to the rapid resistance development against conventional antibiotics with target specific mode of action [9]. The lack of bacterial resistance development towards synthetic amphiphilic polymers have been reported [12], whereas the development of significant bacterial resistance against conventional antibiotic is well documented.

For widespread therapeutic applications, synthetic polymers with high antibacterial activity and concomitant low hemolytic activity and toxicity towards mammalian cells are desired. Research efforts have been focussed to ascertain the effects of various macromolecular structural parameters on the hemolytic and antibacterial activities of synthetic amphiphilic polymers. Structural parameters that have been reported to significantly impact the hemolytic and antibacterial activities include the block *versus* random copolymer architecture [13], amphiphilic balance [14], type of cationic charge [15], facially *versus* segregated amphiphilicity [16], charge density [17], backbone spacer distance [18], and terminal groups [19]. We have reported that the control of spatial charge distribution in polyacrylates with various compositions of 6-carbon and 2-carbon spacer arm (distance between polymer backbone to cationic center) repeat units can lead to polymers with high antibacterial and concomitant low hemolytic activity [20]. In another approach, we investigated the hemolytic and antibacterial activities of polyacrylates (6-carbon spacer arm counits) as a function of polymer amphiphilicity through control of non-ionic hydrophilic poly(ethylene glycol) (PEG) content [21]. The cationic homopolymer with 6-carbon spacer arms PM6-100%, *i.e.*, poly(6-aminohexylacrylate), is highly hemolytic and antibacterial [20,22]. We found that the incorporation of just 33 mol % of poly(ethylene glycol) methyl ether methacrylate comonomer (PEGMA-300) units in this 6-carbon spacer arm polymer led to >1300 times reduction in hemolytic activity while maintaining high antibacterial activity, leading to >100 times selectivity against *Escherichia coli* (*E. coli*) over red blood cells (RBCs) [21]. In these copolymers with long hexyl spacer arms, the cationic charge and hydrophilic moieties are present on the same repeating unit. It would be of importance to understand the effect of polymer structure, in terms of relative placement of cationic and hydrophobic groups, on the impact of PEGylation on hemolytic and antibacterial activities of amphiphilic polymers.

The aim of present investigation is to explore the effect of PEG group incorporation in copolymers having cationic groups and hydrophobic alkyl chains on separate repeating units. These results can be directly compared with our recently synthesized PEGylated 6-carbon spacer arm polymers with cationic and alkyl groups on the same repeating units [21]. Our study revealed that the incorporation of PEG counits has substantially different effects on the antibacterial and hemolytic activities of polymers with topographical differences in the placement of cationic groups relative to hydrophobic groups. The copolymers displayed high bactericidal activity with 5 log reduction of *E. coli* colony forming units within 2 h of treatment with a copolymer.

## 2. Results and Discussion

### 2.1. Synthesis of Copolymers

**Figure 1:**
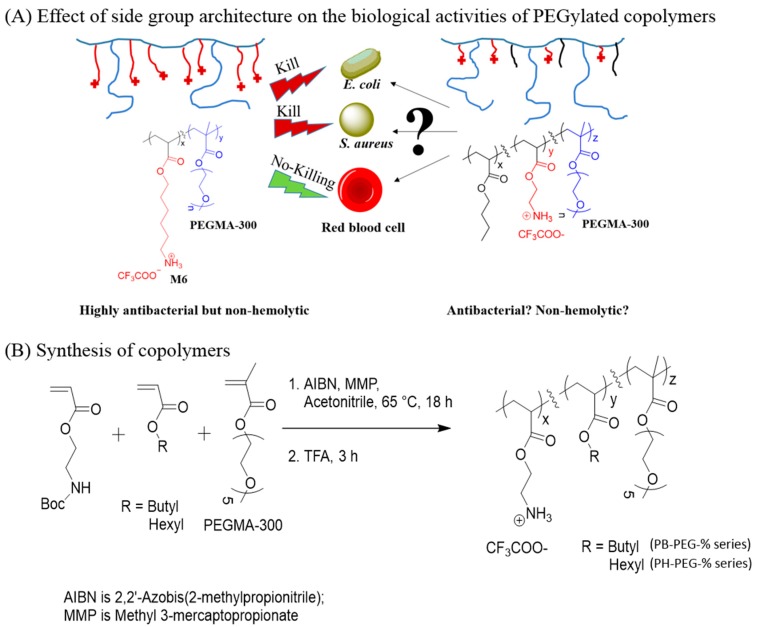
(**A**) Effect of relative topographical positions of cationic and lipophilic groups on the biological activities of PEGylated copolymers (The questions marks in part A signifies that the antibacterial and hemolytic activities of the terpolymer is unknown and the aim of this study was to explore the activities of this terpolymer); and (**B**) Synthesis of cationic amphiphilic copolymers through free radical polymerization.

The copolymers were synthesized through free radical copolymerization of 2-((*tert*-butoxycarbonyl)amino)ethyl acrylate and PEGMA-300 with butyl or hexyl acrylate using 2,2'-azobis(2-methylpropionitrile) (AIBN) as a free radical initiator and methyl 3-mercaptopropionate (MMP) as a chain transfer agent (Figure 1) [20,21]. Two series of copolymers, one with butyl side groups and the other with hexyl side groups were synthesized to explore the effect of copolymer hydrophobicity on the antibacterial and hemolytic activities. The ratio of monomers to AIBN and MMP was kept constant to obtain similar molecular weights for all copolymers. Molecular weights of copolymers were estimated against linear polystyrene standards using gel permeation chromatography and found to be similar for all copolymers (*M*_n_ ~ 4000 g/mol). The feed mole ratio of 2-((*tert*-butoxycarbonyl)amino)ethyl acrylate to hexyl or butyl acrylate was kept constant at 60:40, whereas the feed mole ratio of PEGMA-300 (PEG side groups degree of polymerization (DP)~5) was varied from 0 mol % to 50 mol % in increments of 10 mol %. Copolymers with 75 mol % of PEGMA were also synthesized. The DP of PEG side group was kept at 5 ethylene glycol units as our earlier study has found that PEGMA-300 led to polymers with higher selectivities against bacteria over RBCs, as compared with copolymers incorporating longer PEG side groups (DP~19, PEGMA-950) [21]. The actual feed ratios of polymers were confirmed using ^1^H-NMR spectroscopy and found to be in close agreement with the feed mole ratios (supporting information). For the removal of N-Boc protecting groups, copolymers were treated with excess trifluoroacetic acid. The nomenclature of PB-PEG X% is used to identify the cationic copolymer with butyl acrylate comonomer units and X mol % (in feed) of PEGMA-300 comonomer units. Similarly, PH-PEGX % represents the copolymer with hexyl acrylate and X mol % (in feed) of PEGMA-300 counits.

### 2.2. Antibacterial Activity of Copolymers

**Figure 2:**
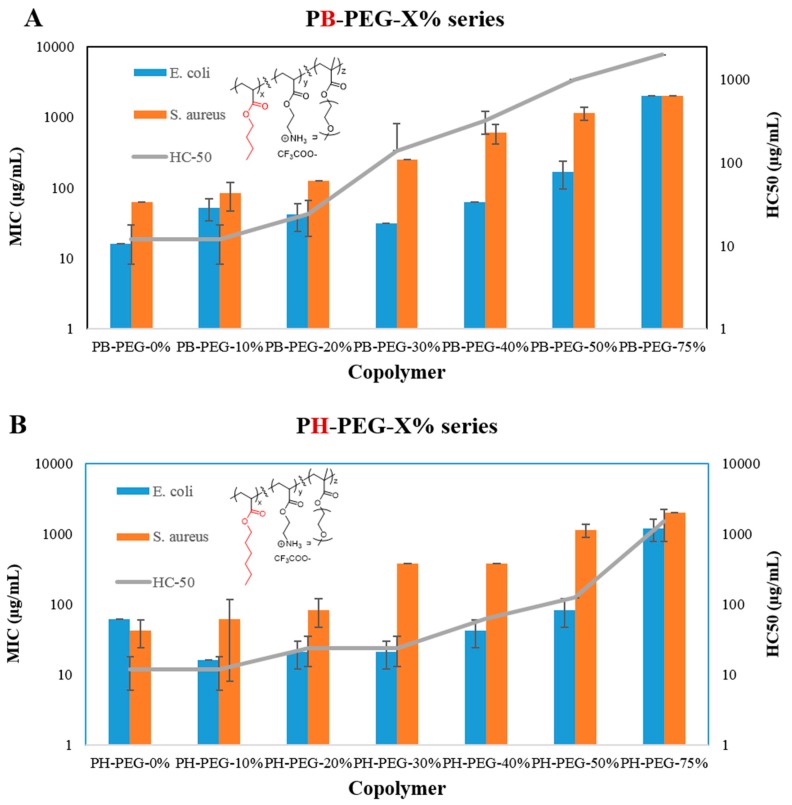
Antibacterial (MIC) and hemolytic activities (HC_50_) of (**A**) PB-PEG-X% series, terpolymers of butyl acrylate (shown in red in structure), 2-((*tert*-butoxycarbonyl)amino)ethyl acrylate, and PEGMA-300; (**B**) PH-PEG-X% series, terpolymers of hexyl acrylate (shown in red in structure), 2-((*tert*-butoxycarbonyl)amino)ethyl acrylate, and PEGMA-300. Error bars represent standard deviation.

The antibacterial activities of copolymers were determined against both gram negative *E. coli* (ampicillin resistant) and the gram positive *Staphylococcus aureus* (*S. aureus*) bacteria in terms of minimum inhibitory concentrations (MIC). MIC is defined as the lowest polymer concentration required to completely inhibit bacterial growth after an incubation period of 18 h. As apparent from Figure 2 and Table 1, PH-PEG-0% displayed high antibacterial activity towards both *E. coli* and *S. aureus*. Kuroda *et al.* have reported high antibacterial activity in polymethacrylate copolymers having high mol % of hydrophobic butyl side groups [23]. As compared with PH-PEG-0%, PH-PEG-10% showed higher activity against *E. coli* (MIC: 16 µg/mL). Addition of small mol % of non-ionic hydrophilic PEG content has been previously reported to enhance the antibacterial activity of *N*-hexylated poly(vinyl pyridines) due to increased wettability in aqueous medium and hence better interactions with the bacterial cell surface [24]. In PH-PEG-0%, high hydrophobicity due to long hexyl side groups can lead to hydrophobic associations of polymer chains in aqueous solution leading to lower number of polymer chains available for interactions with the bacterial cells [23]. Further increase in the mol % of PEGMA-300 repeat units till 50 mol % did not substantially deteriorate the antibacterial activity of these copolymers, but higher than 50 mol % (75 mol %) content led to complete loss of activity against *E. coli* (MIC > 1000 µg/mL). Similar to the effect of PEG on PH copolymer series with hexyl side groups, the addition of PEG side groups did not drastically reduce the antibacterial activities of PB series copolymer until 50 mol % of PEGMA-300 content, and copolymer with 75 mol % was not active against *E. coli*. These observations are similar to the effects of PEG content on the antibacterial activities of PEGylated PM6 copolymers (Table 1) with cationic charge and hydrophobic hexyl spacer arms on the same repeat units [21].

**Table 1:** Characterization and biological activities of amphiphilic polyacrylates.

Copolymer	Alkyl Side Group	Mol % PEGMA-300 (Actual)	*M*_n_ [g/mol] kDa	MIC *E. coli* [µg/mL]	MIC *S. aureus* [µg/mL]	HC_50_ RBC [µg/mL]	Selectivity HC_50_/MIC	
							*E. coli*	*S. aureus*
PB-PEG-0%	Butyl	0%	4.0	16	62	<12	<1	<1
PB-PEG-10%	Butyl	17%	4.2	52	83	<12	<1	<1
PB-PEG-20%	Butyl	23%	3.8	42	125	24	<1	<1
PB-PEG-30%	Butyl	33%	4.1	31	250	140	4.5	<1
PB-PEG-40%	Butyl	44%	4.2	62	604	317	5	<1
PB-PEG-50%	Butyl	53%	4.5	167	1143	1000	6	<1
PB-PEG-75%	Butyl	72%	4.6	2000	2000	2000	1	1
PH-PEG-0%	Hexyl	0%	3.4	62	42	<12	<1	<1
PH-PEG-10%	Hexyl	11%	3.6	16	62	<12	<1	<1
PH-PEG-20%	Hexyl	21%	3.8	21	83	24	1	<1
PH-PEG-30%	Hexyl	27%	3.9	21	384	24	1	<1
PH-PEG-40%	Hexyl	35%	4.6	42	384	62	1.5	<1
PH-PEG-50%	Hexyl	46%	4.6	83	1143	125	1.5	<1
PH-PEG-75%	Hexyl	72%	6.4	1190	2000	1500	1.3	<1
PM6-100 *	Hexyl	0%	5.8	6.5	16	<1.9	<1	<1
PM6-90-PEG300 *	Hexyl	10%	5.1	7.8	31	83	11	2.6
PM6-70-PEG300 *	Hexyl	33%	5.2	16	62	>1809	>113	>29
PM6-50-PEG300 *	Hexyl	50%	5.1	104	250	>2000	>19	>8
PM6-30-PEG300 *	Hexyl	71%	5.9	>1809	>2000	>200	>1	1

* Yang *et al.* [21].

As shown in Figure 2 and Table 1, these PEGylated terpolymers displayed substantially lower activity against gram positive *S. aureus*, as compared with gram negative *E. coli*. Furthermore, increasing the PEGMA-300 content from 0 mol % to 40 mol % led to dramatic reduction in activity against *S. aureus*. Similarly, PEGylated PM6 copolymers (Table 1) previously demonstrated lower activity against *S. aureus* than *E. coli* [21]. These observations are in contrary to other studies that reported higher activities of synthetic amphiphilic polymers (without PEG) against *S. aureus* than *E. coli* [25,26,27]. The double membrane structure of *E. coli* cell surface can be considered more difficult to penetrate than the single membrane cell surface of *S. aureus* [25]. However, dramatically lower activity of these PEGylated copolymers against *S. aureus* than *E. coli* suggests the probable hydrogen bonding associations of long PEG side groups with the thick (15–80 nm) peptidoglycan cell wall of *S. aureus*, thus hindering the permeabilization of PEGylated polymers through the cell surface of *S. aureus*.

### 2.3. Hemolytic Activity of Copolymers

The toxicity of these copolymers against mammalian cells was ascertained in terms of hemolytic activity against freshly drawn mice RBCs. Hemolytic concentration-50% (HC_50_) is defined as the lowest polymer concentration required to lyse 50% of RBCs within an incubation period of 1 h. The hemolytic activity of amphiphilic polymers has been mainly attributed to the permeabilization of polymers into the hydrophobic core of RBC’s lipid bilayer through lipophilic interactions [28]. The outer leaflet of RBCs’ cell membrane lacks net negative charge in contrary to the negatively charged cell surface of bacteria [28]. The incorporation of hydrophilic PEG side groups is expected to reduce the hemolytic activities of synthetic amphiphilic polymers. Similar to the protective action of blood plasma, PEG is believed to hinder the damage to RBCs from the foreign body contact [29]. PEG can weakly adsorb on the surface of RBCs thus further enhancing its protective action [30]. Moreover, the addition of hydrophilic PEG groups can reduce the overall hydrophobicity of polymers and then their insertion through the hydrophobic core of RBC’s lipid bilayer.

However, majority of these PEGylated terpolymers displayed high hemolytic activity (Figure 2 and Table 1). As shown in Table 1, both PM6-100% (HC_50_ < 2 µg/mL) and PB-PEG-0% (HC_50_ < 12 µg/mL) displayed high hemolytic activities. However, incorporation of just 33 mol % of PEGMA-300 led to dramatic reduction in hemolytic activity (HC_50_ > 1809 µg/mL) in PM6-70-PEG300 leading to >113 times selectivity (HC_50_/MIC) against *E. coli* over RBCs. In comparison, PB copolymers showed substantially higher hemolytic activity till 50 mol % of PEG side groups. Thus, the topographical placement of cationic centers relative to hydrophobic alkyl moieties significantly affects the ability of PEG groups to reduce the hemolytic activity of these amphiphilic copolymers. In PM6-70-PEG300, with hexyl groups (as spacer arms) present on the same repeating unit with cationic groups, the addition of PEG dramatically reduced the hemolytic activities of this copolymer. On the other hand, PB-PEG-40% containing butyl and cationic groups (attached to 2-carbon spacer arm) on separate repeat units displayed high hemolytic activity despite the high PEG mol % content. Tew and co-workers have previously reported that “facially amphiphilic” polynorbornenes demonstrated higher selectivities against bacteria over RBCs, as compared with “segregated” polynorbornenes with cationic and alkyl groups on separate repeat units [16]. Similarly, Sen and co-workers have shown higher selectivity of poly(vinyl pyridines) against bacteria over RBCs as compared with separate center copolymers [31]. Our investigations have revealed that the relative positions of cationic and hydrophobic groups along the polymer backbone can have dramatic effect on the ability of PEG groups to protect erythrocytes from the lytic ability of synthetic amphiphilic polyacrylates and these results can have implications in the design of PEGylated polymers for biomedical applications.

PH series copolymers in general displayed higher hemolytic activity than the PB series with shorter butyl side groups. This result is expected due to higher hydrophobicity of copolymers with longer hexyl side groups leading to higher ability of these copolymers to rupture the cell membrane of RBCs through lipophilic interactions.

### 2.4. Time Dependent Killing Efficiency of Copolymers

**Figure 3:**
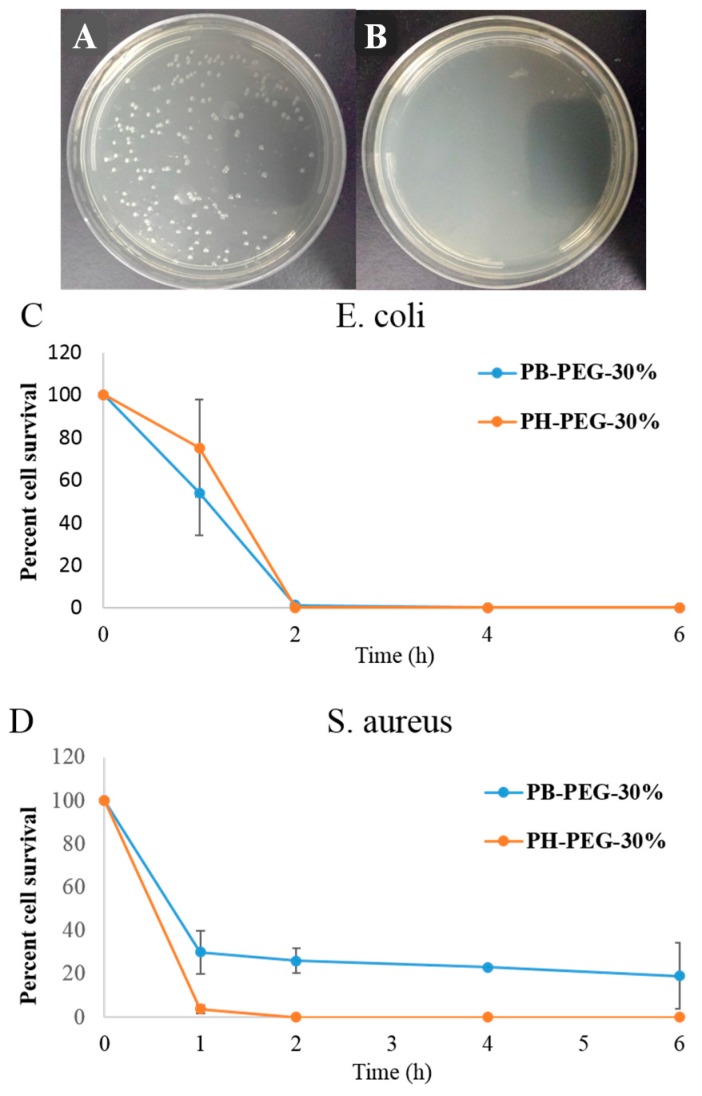
*E. coli* colony forming units (CFUs) after treatment with PB-PEG-30% at (**A**) 0 h and (**B**) at 6 h time interval. Time dependent killing efficiency of PB-PEG-30% and PH-PEG-30% copolymers at 1 × MIC concentration against (**C**) *E. coli* and (**D**) *S. aureus*. PB-PEG-30% and PH-PEG-30% terpolymers incorporate approximately 30 mol % of PEGMA-300. Error bars represent standard deviation.

Time dependent killing efficiencies of PB-PEG-30% and PH-PEG-30% were obtained against *E. coli* and *S. aureus* (Figure 3). Both polymers displayed high bactericidal efficiency against *E. coli* and a 5 log reduction in *E. coli* colony forming units was obtained within 2 h of incubation with 1 × MIC concentration of PH-PEG-30%. These results confirmed the killing activity of these polymers, as opposed to only bacteriostatic activity and indicate that the minimum bactericidal concentration of these polymers against *E. coli* is equal to their minimum inhibitory concentrations.

As discussed above, these PEGylated copolymers demonstrated significantly lower activity against *S. aureus* than *E. coli*. As apparent from Figure 3, a substantial number of *S. aureus* colonies survived after treatment with 1 × MIC concentration of PB-PEG-30% even after 6 h of treatment, whereas more than 98% *E. coli* were killed within 2 h of treatment with PB-PEG-30%. In contrast to PB-PEG-30%, PH-PEG-30% displayed 100% killing against *S. aureus* at 1 × MIC concentration. Longer hexyl groups in PH-PEG-30% can lead to higher cell membrane permeability of polymer chains due to increased hydrophobic interactions with bacterial cell surface. Longer hydrophobic hexyl side groups can lead to more collapsed conformation of polymer chains in the aqueous medium leading to reduced association of PEG groups with the *S. aureus* cell wall.

## 3. Experimental Section

### 3.1. Materials

Ethanolamine, hexyl acrylate, butyl acrylate, 2,2ʹ-azobis(2-methylpropionitrile) (AIBN), methyl 3-mercaptopropionate (MMP), acetonitrile (anhydrous), *N*,*N*-diisopropylethylamine, and tetrahydrofuran (THF) were purchased from Sigma-Aldrich (Milwaukee, WI, USA) and used without further purification. Poly(ethylene glycol) methyl ether methacrylate (PEGMA-300, *M*_n_ ~300 g/mol) and acryloyl chloride were purchased from Sigma-Aldrich. Acryloyl chloride was distilled prior to use and poly(ethylene glycol) methyl ether methacrylate was purified (inhibitor removal) by silica gel column chromatography using dichloromethane as eluent. Trifluoroacetic acid (TFA), di-*tert*-butyl dicarbonate (*t*-Boc), hexane, and diethyl ether were purchased from Alfa Aesar (Ward Hill, MA, USA). Dichloromethane and ethyl acetate were purchased from BDH/VWR (Bridgeport, CT, USA). All other chemicals or reagents were used as obtained without further purification.

### 3.2. Instrumentation

^1^H-NMR spectra of polymers were obtained on a Varian NMR spectrometer (600 MHz, Varian: Palo Alto, CA, USA) using D_2_O as solvent. The molecular weights (*M*_W_, *M*_n_, and polydispersities) of precursor polymers (N-Boc protected) were estimated on an EcoSec HLC-83220 gel permeation chromatography instrument (RI detector, TSKgel SuperHZ-N (3 µm 4.6 mm ID) and TSKgel Super HZ-M (3 µm 4.6 mm ID) columns) by Tosoh Bioscience (Tosoh Bioscience LLC: South San Francisco, CA, USA). Linear polystyrenes were used as standards and THF was used as an eluent at a flow rate of 0.35 mL/min. An Agilent 8453 spectrophotometer (Agilent Technologies, Inc.: Santa Clara, CA, USA) was used to measure bacterial cell growth (using 1 cm path length plastic cuvette) in *E. coli* and *S. aureus* cell cultures. SpectraMax 340 PC microplate reader from Molecular Devices (Molecular Devices: Sunnyvale, CA, USA) was used to obtain OD_595_ (optical density at wavelength of 595 nm) for antibacterial test and OD_414_ for hemolysis test.

### 3.3. N-Boc Protection of Ethanolamine

Eight milliliters (132 mmol) of ethanolamine was added into a 250 mL round bottom flask containing 132 mL deionized water. Thirty milliliters (130 mmol) of di-*tert*-butyl dicarbonate (*t*-Boc) was then dropwise added [20]. The reaction mixture was left under stirring at room temperature for 3 h. N-Boc protected ethanolamine was extracted using ethylacetate (3 × 125 mL) and the organic phase was separated and dried with sodium sulfate. Excess solvent was removed using a rotary evaporator under reduced pressure. The final product was obtained as white powder in 92% yield. ^1^H-NMR (600 MHz, CDCl_3_): δ 1.4 (s, 9H), 3.3 (m, 2H), 3.7 (m, 2H), 5.3 (s, 1H).

### 3.4. Synthesis of 2-((Tert-butoxycarbonyl)amino)ethyl Acrylate Monomer

Fourteen grams (87 mmol) of N-Boc ethanolamine was added in a 500 mL two neck round bottom flask already charged with 23 mL (130 mmol) *N*,*N*-diisopropylethylamine and 130 mL anhydrous dichloromethane [20]. The reaction mixture was closed and purged with nitrogen. A volume of 7.32 mL of acryloyl chloride was added dropwise into the reaction mixture (while stirring) under nitrogen atmosphere at 0 °C. The reaction mixture was allowed to warm to room temperature and left under stirring for 18 h. The reaction mixture was then washed with deionized water (3 × 125 mL), 10% citric acid (3 × 125 mL), 10% potassium carbonate solution (3 × 125 mL), and saturated sodium bicarbonate solution (3 × 125 mL). The organic phase was separated and dried with sodium sulfate. Excess solvent was removed under reduced pressure using the rotary evaporator. The resultant liquid was purified by column chromatography using ethyl acetate/hexane (1:1) mixture as eluent. 65% yield. ^1^H-NMR (600 MHz, CDCl_3_): 1.40 (s, 9H), 3.47 (s, 2H), 4.22 (s, 2H), 5.83 (d, 1H), 6.10 (q, 1H), 6.39 (d, 1H).

### 3.5. Synthesis of Copolymers

A representative synthetic procedure is as follows [20,21]. 2-((*tert*-butoxycarbonyl)amino)ethyl acrylate (0.387 g, 1.8 mmol), 0.187 g (1.2 mmol) hexyl acrylate, and 0.900 g (3 mmol) PEGMA-300 were added into a 100 mL three-neck round bottom flask which was already charged with 9.85 mg (0.06 mmol) AIBN, 33.3 µL MMP, and 6 mL anhydrous acetonitrile. The flask was sealed and degassed with nitrogen (using stainless steel needle) for 20 min. The reaction mixture was then stirred for 18 h at 65 °C. Afterwards, acetonitrile in the solution was removed under reduced pressure using the rotary evaporator (Buchi Analytical Inc.: New Castle, PA, USA). The polymer was dissolved in THF and precipitated in hexane three times. In order to remove N-Boc protecting groups, the polymer was dissolved in excess TFA and the solution was stirred at room temperature for 3 h. Afterwards the TFA was removed under reduced pressure using the rotary evaporator and the polymer was dissolved in methanol and precipitated in diethyl ether multiple times. Resultant polymer was kept under vacuum for 3 days and lyophilized. Yield 0.82 g.

### 3.6. Antibacterial Activity Test

Antibacterial activities of cationic amphiphilic copolymers were determined against *E. coli* (TOP 10, ampicillin resistant) and *S. aureus* ATCC 25923 using a microdilution assay as described in the literature [23]. Stock solutions (20 mg/mL) of polymers were prepared by dissolving each polymer in dimethyl sulfoxide. To obtain different concentrations, serial dilutions and some intermediate dilutions were prepared by further adding deionized water. Control solutions (without polymer) were similarly prepared. *E. coli* bacteria were incubated overnight at 37 °C, under shaking, in culture tubes containing Luria Bertani (LB) broth and ampicillin. Bacterial growth was measured with an Agilent 8453 spectrophotometer (Agilent Technologies, Inc.: Santa Clara, CA, USA) using a disposable plastic cuvette with a path length of 1 cm. The device measures turbidity at λ = 595 nm (optical density, OD_595_). The bacterial culture was diluted with fresh LB to achieve the OD_600_ = 0.1 of cell suspension and this suspension was incubated at 37 °C for approximately 90 min. The OD_595_ value was increased to 0.5, indicating the log-phase of exponential growth. The culture was then diluted with fresh LB broth to obtain a final OD_595_ value of 0.001. Polymers or control solutions were added to the 96 well cell culture plates followed by the addition of final *E. coli* cell suspension and the plates were incubated at 37 °C for 18 h to promote bacterial growth. Bacterial growth was measured on a SpectraMax 340 PC microplate reader, using turbidity at λ = 595 nm (OD_595_). Antibacterial activity of polymers was similarly determined against *S. aureus*, except Mueller-Hinton (MH) broth was used in place of LB broth and longer log phase time intervals were required. Minimum inhibitory concentration (MIC) is defined as the lowest polymer concentration required to completely inhibit the bacterial growth after an incubation period of 18 h. MIC values reported here are the averages of three independent experiments on separate days.

### 3.7. Hemolytic Activity Test

Hemolytic activity of copolymers was tested against mice red blood cells (RBCs) [23]. The RBCs were obtained from centrifuging the freshly drawn blood at 3000 rpm for 15 min. Plasma and white blood cells were removed as supernatant. RBCs were washed with TBS (Tris-buffered saline; 10 mM Tris, 150 mM NaCl, at pH = 7) solution. The RBCs were 40-fold diluted with TBS solution to prepare a final 0.25% RBC stock solution. The stock solution (120 µL), polymer solution (15 µL) or control solution, and TBS solution (15 µL) were added to 600 µL micro centrifuge tubes and incubated at 37 °C for 1 h. The tubes were then centrifuged for 5 min at 4000 rpm. The resultant supernatant (30 µL, in triplicate) and 70 µL TBS were added in each well of the 96 well tissue culture plates. Hemoglobin concentration was measured as OD_414_, and the percent hemolysis corresponding to each polymer concentration was calculated using the following formula:

(1) $$\%\;Hemolysis=\;\left(\frac{OD_{414}Polymer-OD_{414}negative\;Control}{OD_{414}Triton-OD_{414}negative\;Control}\right)\times 100$$

Hemolytic activity-50% (HC_50_) is defined as the lowest polymer concentration required to cause 50% lyses in RBCs within an incubation period of 1 h. The HC_50_ values reported here are the averages of two independent experiments performed on separate days.

### 3.8. Time Dependent Killing Efficiency Test

The time dependent killing efficiencies of polymers were obtained as per reported procedure [21]. *E. coli* and *S. aureus* bacterial cell cultures were obtained in log phase growth as described above in the antibacterial test section. A final stock solution with OD_595_ = 0.001 (~10^5^ CFU/mL) was obtained by diluting the log phase cell suspension with fresh nutrient broth (LB broth for *E. coli* and MH broth for *S. aureus*). This final stock cell suspension was treated with polymer solutions (1 × MIC final concentration) or deionized water (without polymer) as control. At regular time intervals (1, 2, 4, and 6 h) the bacterial cell suspension samples were taken out. After serial dilutions (in 10 folds), 20 µL of the final dilution was streaked on agar plates and left for incubation for 24 h at 37 °C. The colony forming units were counted using the image J image processing and analysis software (National Institute of Health: Bethesda, MD, USA). Results shown here are the averages of 2 independent experiments performed on separate days.

## 4. Conclusions

In conclusion, PEGylated cationic amphiphilic acrylate copolymers with high antibacterial activity against *E. coli* and *S. aureus* were synthesized. Time-kill studies revealed a 5 log reduction in *E. coli* CFUs/mL within 2 h of polymer treatment. As compared with PB-PEG-30%, PH-PEG-30% with two carbon longer alkyl side group displayed higher bactericidal activity against *S. aureus* with 100% killing of *S. aureus* cells within 2 h. The PEGylated copolymers reported here demonstrated substantially lower activity against *S. aureus* than *E. coli*. The hydrogen bonding association of hydrophilic PEG groups with peptidoglycans in the thick cell wall of *S. aureus* can be a contributing factor to this observation. The effect of non-ionic hydrophilic modifications on the antibacterial and hemolytic activities of amphiphilic polyacrylates was investigated. A significantly different impact of PEGylation on the hemolytic activities of polymers with variation in relative positions of cationic and hydrophobic centers was observed. As reported earlier, in copolymers with cationic centers and hydrophobic units (hexyl spacer arms) on the same repeat units, the incorporation of PEGMA-300 counits dramatically reduced the hemolytic activity without affecting antibacterial activity, as compared with the homopolymer PM6-100% lacking PEG groups. Similar to the high hemolytic activity of PM6-100%, PB-PEG-0% without PEG content and with cationic and alkyl groups present on separate repeat units showed high hemolytic and antibacterial activities. However, the incorporation of PEGMA-300 did not substantially mitigate the hemolytic activity of these copolymers even at a high mol % (40%) content of PEGMA-300. These results indicate that the PEG’s protective effect on RBCs is greatly influenced by the topographical placements of cationic centers and hydrophobic groups along the polymer chain. A PEGylated polymer architecture with long hydrophobic spacer arms for pendent cationic groups can result in high antibacterial and simultaneous low toxicity towards mammalian cells.

## Supplementary Materials

Supplementary materials can be found at http://www.mdpi.com/1422-0067/16/10/23867/s1.
