# Supplementary material for: Effect of Relative Arrangement of Cationic and Lipophilic Moieties on Hemolytic and Antibacterial Activities of PEGylated Polyacrylates
Source: Int J Mol Sci. 2015 Oct 9;16(10):23867–80. doi: 10.3390/ijms161023867 (PMC4632729; doi:10.3390/ijms161023867)
Supplement: Supplementary file 1 [file ijms-16-23867-s001.docx]

Supplementary Information

**^1^H-NMR Spectra of Copolymers**

**PH-PEG-0% ^1^H-NMR (600 MHz, D_2_O):** δ 0.7–0.9 (bm, 14H), 1.15–1.37 (bm, 30H), 1.4–2.5
(bm, 51H), 2.51–2.7 (m, 4H), 3.1–3.3 (bm, 17H), 3.45–3.6 (m, 3H), 3.8–4.3 (bm, 26H).

**PH-PEG-10% ^1^H-NMR (600 MHz, D_2_O):** δ 0.7–0.9 (bm, 12H), 0.91–1.15 (m, 5H), 1.16–1.37
(bm, 24H), 1.4–2.5 (bm, 45H), 2.52–2.75 (m, 4H), 3.1–3.35 (bm, 18H), 3.4–3.7 (bm, 32H), 3.85–4.3 (bm, 17H).

**PH-PEG-20% ^1^H-NMR (600 MHz, D_2_O):** δ 0.7–1.15 (bm, 21H), 1.17–1.4 (bm, 24H), 1.45–2.5
(bm, 44H), 2.52–2.8 (bm, 4H), 3.05–3.3 (bm, 23H), 3.4–3.75 (bm, 52H), 3.8–4.3 (bm, 25H).

**PH-PEG-30% ^1^H-NMR (600 MHz, D_2_O):** δ 0.7–1.13 (bm, 27H), 1.15–1.4 (bm, 25H), 1.5–2.5
(bm, 44H), 2.52–2.8 (m, 4H), 3.1–3.4 (bm, 28H), 3.47–3.7 (bm, 79H), 3.8–4.3 (bm, 28H).

**PH-PEG-40% ^1^H-NMR (600 MHz, D_2_O):** δ 0.7–1.15 (bm, 36H), 1.16–1.4 (bm, 30H), 1.15–2.5
(bm, 54H), 2.51–2.75 (m, 4H), 3.17–3.3 (bm, 39H), 3.5–3.7 (bm, 127H), 3.8–4.3 (bm, 40H).

**PH-PEG-50% ^1^H-NMR (600 MHz, D_2_O):** δ 0.7–1.16 (bm, 67H), 1.16–1.4 (bm, 44H), 1.5–2.5
(bm, 80H), 2.51–2.7 (m, 4H), 3.1–3.3 (bm, 65H), 3.4–3.73 (bm, 248H), 3.8–4.3 (bm, 62H).

**PH-PEG-75% ^1^H-NMR (600 MHz, D_2_O):** δ 0.7–1.14 (bm, 81H), 1.2–2.5 (bm, 94H), 2.51–2.7
(bm, 4H), 3.1–3.3 (bm, 77H), 3.4–3.8 (bm, 346H), 3.9–4.3 (bm, 65H).

**PB-PEG-0% ^1^H-NMR (600 MHz, D_2_O):** δ 0.75–0.9 (bm, 13H), 1.2–1.34 (bm, 10H), 1.35–2.5
(bm, 51H), 2.51–2.7 (m, 4H), 3.1–3.25 (bm, 18H), 3.5–3.6 (m, 3H), 3.9–4.3 (bm, 26H).

**PB-PEG-10% ^1^H-NMR (600 MHz, D_2_O):** δ 0.7–0.9 (bm, 16H), 0.91–1.15 (bm, 5H), 1.2–2.5
(bm, 66H), 2.51–2.7 (m, 4H), 3.1–3.25 (bm, 22H), 3.48–3.7 (bm, 27H), 3.9–4.3 (bm, 30H).

**PB-PEG-20% ^1^H-NMR (600 MHz, D_2_O):** δ 0.78–1.17 (bm, 20H), 1.2–2.5 (bm, 51H), 2.52–2.75
(m, 4H), 3.1–3.3 (bm, 25H), 3.5–3.7 (bm, 45H), 3.9–4.3 (bm, 26H).

**PB-PEG-30% ^1^H-NMR (600 MHz, D_2_O):** δ 0.76–1.14 (bm, 22H), 1.2–2.5 (bm, 48H), 2.52–2.75
(m, 4H), 3.1–3.3 (bm, 28H), 3.4–3.7 (bm, 57H), 3.9–4.3 (bm, 26H).

**PB-PEG-40% ^1^H-NMR (600 MHz, D_2_O):** δ 0.7–1.15 (bm, 22H), 1.21–2.5 (bm, 42H), 2.51–2.75
(m, 4H), 3.1–3.3 (bm, 31H), 3.42–3.72 (bm, 68H), 3.8–4.3 (bm, 25H).

**PB-PEG-50% ^1^H-NMR (600 MHz, D_2_O):** δ 0.7–1.16 (bm, 35H), 1.2–2.5 (bm, 77H), 2.51–2.76
(m, 4H), 3.1–3.3 (bm, 34H), 3.4–3.7 (bm, 146H), 3.8–4.3 (bm, 34H).

**PB-PEG-75% ^1^H-NMR (600 MHz, D_2_O):** δ 0.7–1.2 (bm, 74H), 1.2–2.5 (bm, 76H), 2.5–2.73
(m, 4H), 3.1–3.3 (bm, 69H), 3.46–3.75 (bm, 377H), 3.8–4.3 (bm, 58H).

Calculation of Actual mole% of Comonomers from ^1^H-NMR

Actual mole contents were calculated through analysis of ^1^H-NMR spectra. Using PH-PEG-30% as an example, the integrations of the peaks of interest for mole percentage calculation are positioned at:
4.0 ppm (hexyl acrylate’s methylene proton), 3.3 ppm (PEGMA-300’s methyl proton), and 3.2 ppm
(2-aminoethyl acrylate’s methylene proton). The formula for the sum of integrations after content adjustments is as follows:

(3.40/2) + (6.46/3) + (5.41/2) = 6.56

The mole contents of each comonomer can be found by dividing the adjusted integrations of the comonomer by the sum of adjusted integrations of all three comonomers.

Hexyl acrylate mole content: [(3.40/2)/6.56] × 100% = 26%

PEGMA mole content: [(6.46/3)/6.56] × 100% = 33%

2-Aminoethyl acrylate mole content: [(5.41/2)/6.56] × 100% = 41%


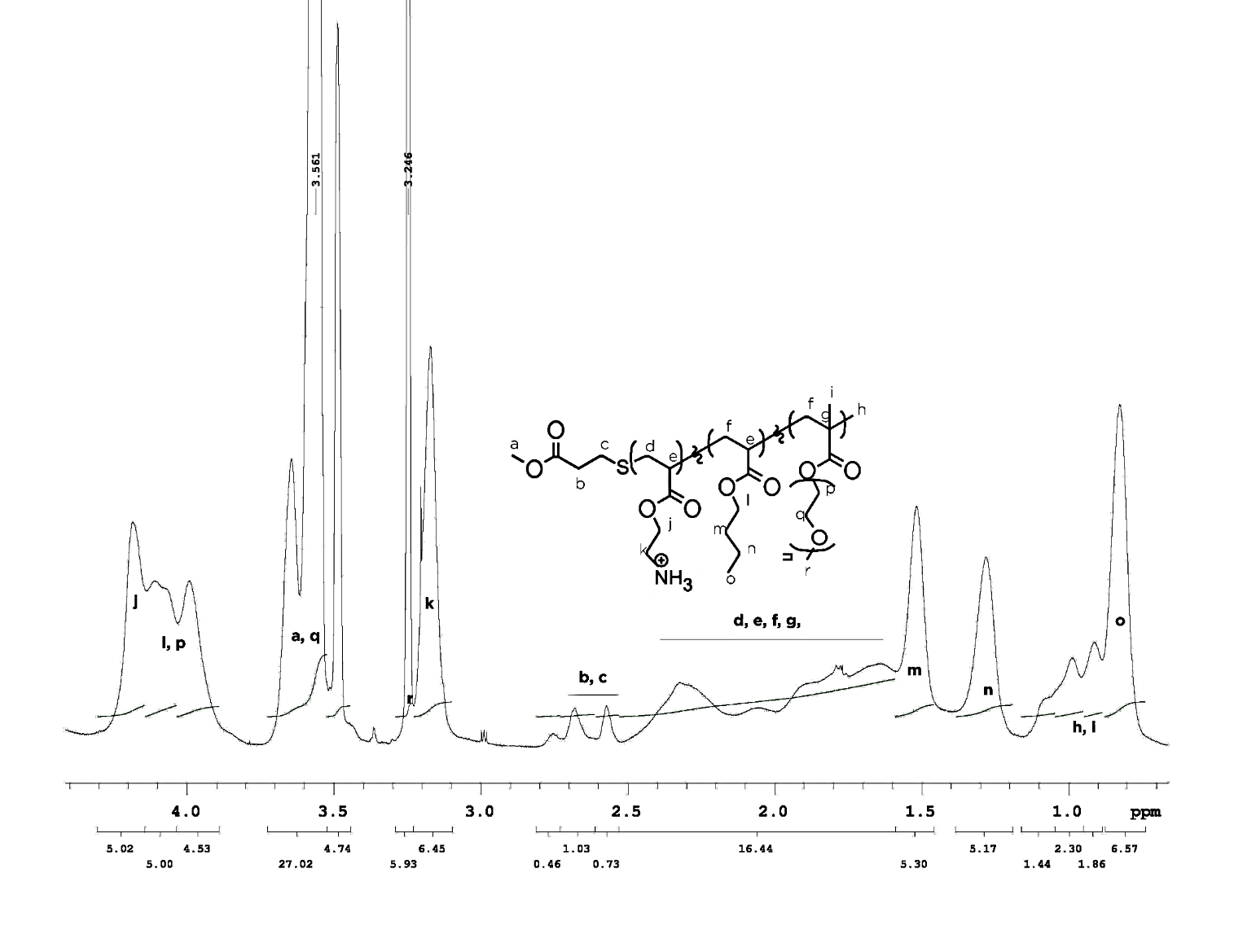


**Figure S1.** ^1^H-NMR of PH-PEG-30% (42% M2, 28% butyl acrylate, 30% PEG).

**Table S1.** Characterization of copolymers.

| **Copolymer** | **Alkyl Side Group** | ***M*_w_ kDa** | ***M*_n_ kDa** | **PDI** | **Actual Mol % Hydrophobic  Monomer (Butyl or Hexyl Acrylate)** | **Mol % 2-((*Tert*-butoxycarbonyl) amino)ethyl Acrylate (Actual)** |
| --- | --- | --- | --- | --- | --- | --- |
| PB-PEG-0% | Butyl | 5.1 | 4.0 | 1.28 | 36.5 | 63.5 |
| PB-PEG-10% | Butyl | 5.6 | 4.2 | 1.34 | 33.3 | 55.7 |
| PB-PEG-20% | Butyl | 5.1 | 3.8 | 1.35 | 31.3 | 47.9 |
| PB-PEG-30% | Butyl | 5.8 | 4.1 | 1.41 | 30.3 | 43.2 |
| PB-PEG-40% | Butyl | 5.9 | 4.2 | 1.41 | 28.7 | 36.5 |
| PB–PEG-50% | Butyl | 6.6 | 4.5 | 1.36 | 22.8 | 30.8 |
| PB–PEG-75% | Butyl | 7.1 | 4.6 | 1.54 | 13.9 | 14.6 |
| PH-PEG-0% | Hexyl | 4.4 | 3.4 | 1.28 | 35 | 65 |
| PH-PEG-10% | Hexyl | 4.6 | 3.6 | 1.28 | 31 | 52 |
| PH-PEG-20% | Hexyl | 5.2 | 3.8 | 1.36 | 31 | 46 |
| PH-PEG-30% | Hexyl | 5.2 | 3.9 | 1.34 | 26 | 41 |
| PH-PEG-40% | Hexyl | 6.7 | 4.6 | 1.46 | 23 | 33 |
| PH-PEG-50% | Hexyl | 7.2 | 4.6 | 1.57 | 18 | 29 |
| PH-PEG-75% | Hexyl | 9.9 | 6.4 | 1.59 | 12 | 16 |
